# Evaluation of Multilevel Surgeries in Children With Spastic Cerebral Palsy Based on Surface Electromyography

**DOI:** 10.3389/fnins.2021.680645

**Published:** 2021-07-15

**Authors:** Sujiao Li, Xueqin Luo, Song Zhang, Yuanmin Tang, Jiming Sun, Qingyun Meng, Hongliu Yu, Chengyan Sun

**Affiliations:** ^1^Institute of Rehabilitation Engineering and Technology, School of Medical Device and Food Engineering, University of Shanghai for Science and Technology, Shanghai, China; ^2^Shanghai Engineering Research Center of Assistive Devices, University of Shanghai for Science and Technology, Shanghai, China; ^3^Department of Functional Neurosurgery, Shanghai Punan Hospital, Shanghai Eber Medical Group, Shanghai, China; ^4^Department of Pediatric Neurosurgery, Xinhua Hospital, Shanghai JiaoTong University School of Medicine, Shanghai, China; ^5^College of Rehabilitation Sciences, Shanghai University of Medicine and Health Sciences, Shanghai, China

**Keywords:** sEMG signal, muscle activity, cerebral palsy, gait analysis, multilevel surgery

## Abstract

The root mean square (RMS) of the surface electromyography (sEMG) signal can respond to neuromuscular function, which displays a positive correlation with muscle force and muscle tension under positive and passive conditions, respectively. The purpose of this study was to investigate the changes in muscle force and tension after multilevel surgical treatments, functional selective posterior rhizotomy (FSPR) and tibial anterior muscle transfer surgery, and evaluate their clinical effect in children with spastic cerebral palsy (SCP) during walking. Children with diplegia (*n* = 13) and hemiplegia (*n* = 3) with ages from 4 to 18 years participated in this study. They were requested to walk barefoot at a self-selected speed on a 15-m-long lane. The patient's joints' range of motion (ROM) and sEMG signal of six major muscles were assessed before and after the multilevel surgeries. The gait cycle was divided into seven phases, and muscle activation state can be divided into positive and passive conditions during gait cycle. For each phase, the RMS of the sEMG signal amplitude was calculated and also normalized by a linear envelope (10-ms running RMS window). The muscle tension of the gastrocnemius decreased significantly during the loading response, initial swing, and terminal swing (*p* < 0.05), which helped the knee joint to get the maximum extension when the heel is on the ground and made the heel land smoothly. The muscle force of the gastrocnemius increased significantly (*p* < 0.05) during the mid-stance, terminal stance, and pre-swing, which could generate the driving force for the human body to move forward. The muscle tension of the biceps femoris and semitendinosus decreased significantly (*p* < 0.05) during the terminal stance, pre-swing, and initial swing. The decreased muscle tension could relieve the burden of the knee flexion when the knee joint was passively flexed. At the terminal swing, the muscle force of the tibial anterior increased significantly (*p* < 0.05), which could improve the ankle dorsiflexion ability and prevent foot drop and push forward. Thus, the neuromuscular function of cerebral palsy during walking can be evaluated by the muscle activation state and the RMS of the sEMG signal, which showed that multilevel surgical treatments are feasible and effective to treat SCP.

## Introduction

Cerebral palsy (CP) refers to a group of persistent motor and postural developmental disorder syndrome that leads to restricted mobility, which is caused by non-progressive brain damage to the developing fetus or infant (Bell et al., 2002). Spastic cerebral palsy (SCP) is the most common type of CP, accounting for ~60–70% of all children with CP. SCP represents a series of neurofunctional disorders, involving joint stiffness, decreased physical activity, tendon hyperreflexia, strong flexor reflex, and strong resistance when muscle is passively stretched (Gage and Novacheck, 2001). Many studies have shown that the gait cycle of patients with SCP presented an abnormal pattern (Winters et al., 1987; Perry and Davids, 1992; Crenna, 1998). Information on different gait patterns could improve early treatment in children with bilateral CP before abnormal gait patterns are fully established (Domagalska–Szopa and Szopa, 2019). Abnormal gait not only affects the patient's joints but also modifies muscle activity and activation patterns (Patikas et al., 2005).

Significant progress has been made in the treatments for CP, especially SCP. To date, the clinical methods for treating SCP mainly include functional training, surgical treatment, physical therapy, acupuncture and massage, and drug therapy (Chin et al., 2020). Comparing these methods, surgical treatment is a very efficient method for patients with severe movement disorders (Buddhdev et al., 2017). The key purpose of surgery is to adjust muscle tension and balance muscle force. In order to adjust the muscle tension of the patient, neurosurgery is mainly performed on the patient such as selective posterior rhizotomy (SPR), which uses an electrophysiological equipment to monitor the electromyogram of multiple muscles of the limbs during the operation and choose continuous recording of somatosensory evoked potentials as an objective basis for the proportion of surgical resection (Turner, 2009). Thus, the muscle tension of the patients can be adjusted fully (Graham et al., 2016; Qijia et al., 2019). In recent years, functional selective posterior rhizotomy (FSPR) has been developed on the basis of SPR. The treatment technologies have risen from the anatomical level to the functional level, and it can regulate muscle tension more effectively. Orthopedic surgery is generally used to balance muscle force. Kapti (2014) utilized the posterior tibial muscle transfer method to treat foot drop, and Fox et al. (2009) solved knee stiffness by rectus femoris transfer surgery. Some studies showed that tibial anterior muscle transfer can treat clubfoot (El-Fadl and Mahmoud, 2013; El Batti et al., 2016; Agarwal et al., 2020a). In general, surgeons perform compound surgeries on patients for some specific malfunctions to adjust muscle tension and balance muscle force. With the increasing number of treatment options, the evaluation of surgical effect has become a very important work.

The surface electromyography (sEMG) signal has been proven to be a reliable reflection of the muscles in the gait of patients with CP (Granata et al., 2005; Patikas et al., 2005; Nardo et al., 2019; Parent et al., 2019). sEMG signal is important in clinical evaluation and rehabilitation medicine with specific focus on neurorehabilitation (Campanini et al., 2020; Cappellini et al., 2020). In recent years, there have been more and more researches focused on the change of sEMG signals for patients with CP after surgical treatments. Some studies have demonstrated that surgery can affect sEMG signals of patients with CP (Patikas et al., 2007). It was proven that the semitendinosus activation timing was delayed and the burst duration of the vastus lateralis was decreased after surgery (Buurke et al., 2004). Lauer et al. (2007) have also proven time–frequency changes of the sEMG signal after hamstring lengthening in children with CP. Wang et al. (2011) reported that EMG signals have changed significantly after selective femoral neurotomy, which deduced that surgery could reduce the muscle tension of the quadriceps muscle. These studies suggested that muscle tension could be reflected by the root mean square (RMS) of sEMG signal. At the same time, studies have shown that the RMS of the sEMG signal is a reliable parameter (Farina et al., 2004) and displayed a positive correlation with muscle force and muscle tension under positive and passive conditions (Onishi et al., 2000). In a complete gait cycle, the activation states of sEMG signals in different subphases are varied (Perc, 2005). However, there are few studies on the muscle force and muscle tension of patients with CP during walking.

In this study, we analyzed the treatment of patients undergoing both FSPR and tibial anterior muscle transfer surgeries. The overall aim of this article was to evaluate the neuromuscular function of CP during walking by the muscle activation state and the RMS of the sEMG signal. By analyzing the changes of the RMS in each subphase, the outcome showed that the patient's muscle force increased and muscle tension decreased after multilevel surgeries. It implies that the neuromuscular function has been improved greatly.

## Methods

### Subjects

Sixteen patients with CP who underwent orthopedic surgery from July 2019 to May 2020 were enrolled in this study. These subjects consisted of 13 cases of diplegia and three cases of hemiplegia in 10 male patients and six female patients, aged 4–17 years (mean age, 9.8 ± 5.0 years). The clinical data of examined patients are shown in Table 1. The muscle tension of the knee and ankle joints on the sagittal plane was tested separately by the Modified Ashworth Scale (MAS). The higher the grade, the higher the abnormal muscle tension in children with CP, and grade 0 represents normal muscle tension. In the clinic, doctors use the Manual Muscle Testing (MMT) to detect the muscle force level of patients. Level 5 is the highest level and represents normal muscle force. As the level decreases, the muscle force decreases. The main manifestations of patients were crouching gait and jumping gait. These two symptoms are specifically manifested as abnormal knee flexion, limited ankle dorsiflexion, and foot varus during walking. No subject had received any treatment (surgery, orthopedics, or Botox injection) before the test, and all were able to walk independently without assistance.

**Table 1:** Demographic and clinical data of the patients.

			**Modified Ashworth Scale (MAS)**				**Manual Muscle Testing (MMT)**			
**ID**	**Involved side**	**Age (years)**	**KE**	**KF**	**AD**	**AP**	**Qua**	**Ham**	**Tib**	**Gas**
1	Right	6–18	3	2	3	2	3	3	2	2
2	Right	3–6	2	2	3	2	3	3	3	3
3	Right	6–18	2	2	3	2	3	3	2	2
4	Both	6–18	3	2	3	2	3	3	2	2
5	Both	6–18	2	2	3	2	3	3	3	3
6	Both	6–18	2	2	3	2	4	4	4	4
7	Both	6–18	2	2	3	2	4	3	3	3
8	Both	3–6	1	1	3	1	4	3	3	3
9	Both	3–6	2	2	3	2	4	4	3	3
10	Both	3–6	2	2	3	2	4	3	3	3
11	Both	6–18	2	2	3	2	3	3	2	2
12	Both	3–6	2	2	3	2	4	3	3	3
13	Both	6–18	2	2	1	2	4	3	3	3
14	Both	6–18	1	1	3	1	4	3	4	4
15	Both	3–6	2	2	3	2	4	3	3	3
16	Both	6–18	2	2	3	2	4	4	4	4

*KE, knee extension; KF, knee flexion; AD, ankle dorsiflexion; AP, ankle plantar flexion; Qua, quadriceps femoris; Ham, hamstring; Tib, tibialis anterior; Gas, gastrocnemius*.

### Procedure and Instruments

**Figure 1:**
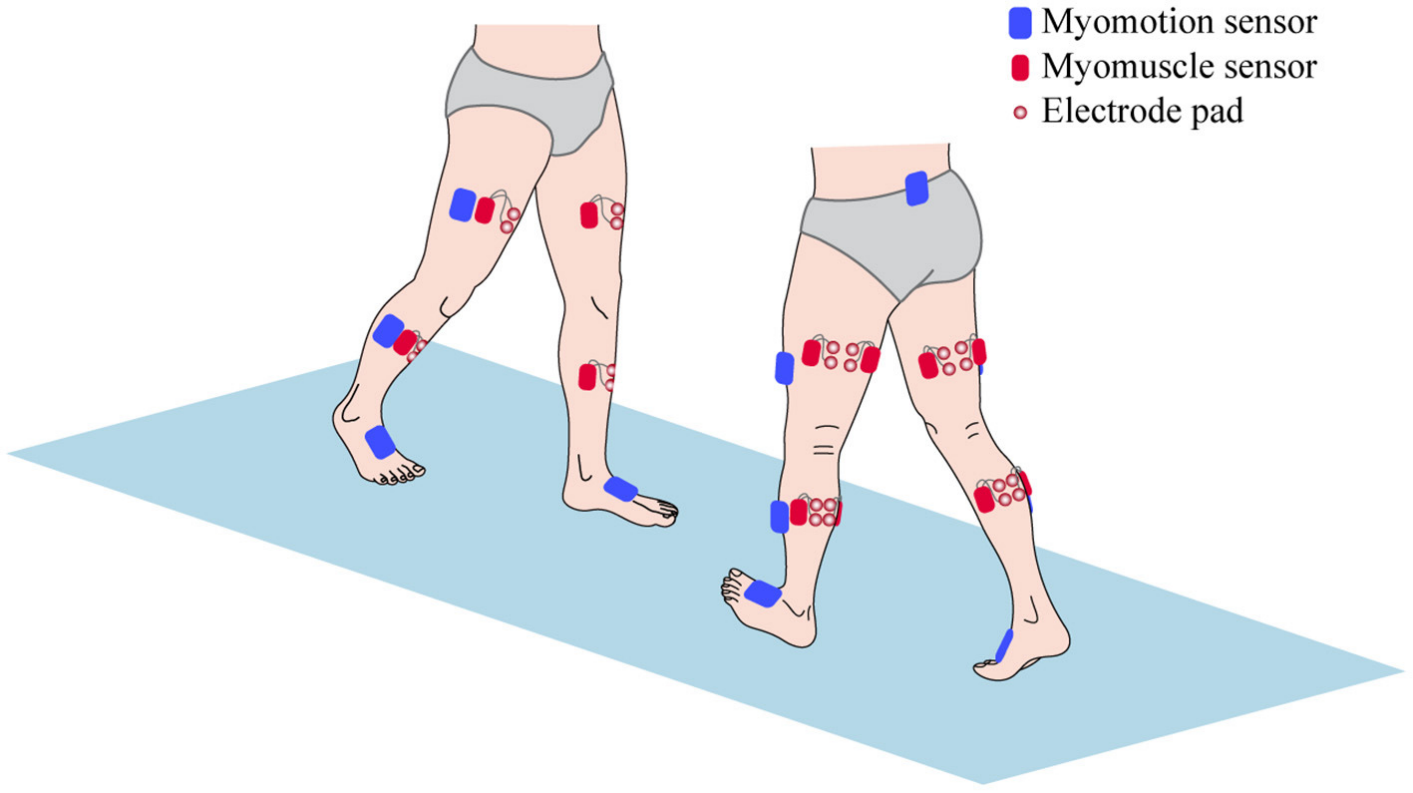
The sensors setup of surface electromyography (sEMG) signal and kinetic parameters collection.

All patients were carried out to collect kinetic parameters and sEMG signals during a gait cycle. The Motion Analysis (NORAXON Inc., Scottsdale, AZ, USA) including Myomotion and Myomuscle module was used to synchronously collect dynamic joint angle and sEMG signals, respectively. The sample frequency of the Myomuscle module is 1,500 Hz. All sensors are wireless, which are simple and light to wear and reduce the impact on the original gait of patients. The electrode pads have been applied over the respective muscles with an interelectrode distance of 2 cm. The direction of these two test electrodes was parallel to the direction of the long axis of the test muscle fiber, and then the corresponding sensors were fixed (Figure 1). After all subjects have put on the equipment, they first perform short exercises to adapt to their own walking rhythm. When the test started officially, the subjects were asked to walk barefoot at a self-selected speed on a 15-m-long lane.

Kinematic parameters were recorded in the sagittal, coronal, and transverse planes for the hip, knee, and ankle to document the preoperative and postoperative status of the patients. For reasons of simplicity, the presentations of the kinematic parameters were focused on the hip and knee joint on the sagittal plane and the ankle joint on the sagittal plane and the coronal plane. The sEMG signals of some muscles were simultaneously recorded. Six muscles were selected as representatives for the knee and ankle. The measured muscles included thigh muscles: rectus femoris, biceps femoris, semitendinosus; and calf muscles: tibialis anterior, lateral gastrocnemius, and medial gastrocnemius.

### Signal Analysis

For each of the following subphases of a gait cycle: loading response, mid-stance, terminal stance, pre-swing, initial swing, mid-swing, and terminal swing, the averaging of all strides for each side, the sEMG signal, and joint angles were calculated separately. In order to reduce the measurement error, six gait cycles for each dependent variable and condition were calculated. The definition of these subphases was made according to the foot strike and foot off of both feet (Perry and Davids, 1992). The kinematics data of the knee and ankle joints were analyzed mainly in order to analyze the surgical effects of the crouching gait and clubfoot. The crouching gait of the knee joint and the clubfoot were reflected mainly in the knee angle of the sagittal plane and the ankle angle of the sagittal plane and coronal plane, respectively.

**Figure 2:**
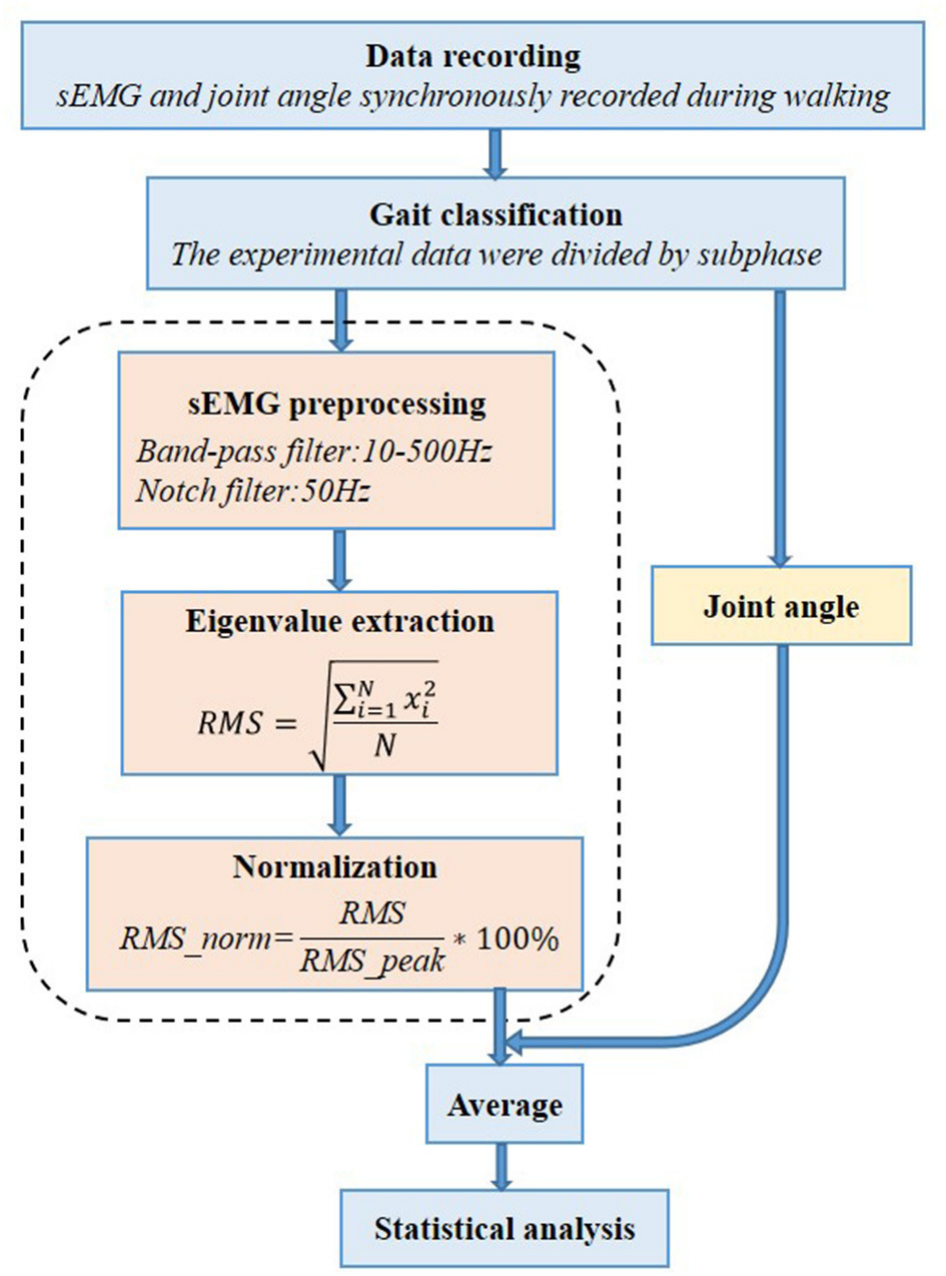
Processing procedure of the surface electromyography (sEMG) signal data analysis.

The raw sEMG signal data were band-pass filtered using a Butterworth filter between 10 and 500 Hz to remove non-EMG artifacts. We also applied a 50-Hz notch filter to remove the power line interference (Daly et al., 2019). The sEMG signal amplitude is affected by several other factors; to adjust for this variability and allow comparison between participants, the sEMG signal is usually normalized to a standard value, usually the peak value of the sEMG signal obtained during the maximum voluntary isometric contraction (MVIC). But for the children with CP, it may be difficult to perform MVIC because it is a challenge to automatically generate the MVIC. In this case, it is considered a feasible and appropriate method to normalize the sEMG signal obtained in a specific task (such as walking) to the peak value. For each percentage of all sEMG signal channels and gait periods, the RMS of the sEMG signal was calculated with a 10-ms running window. For each sEMG signal channel, the highest RMS value (peak RMS) was obtained and used for normalization (Gagnat et al., 2020). The procedure of sEMG signal data processing is shown in Figure 2.

**Figure 3:**
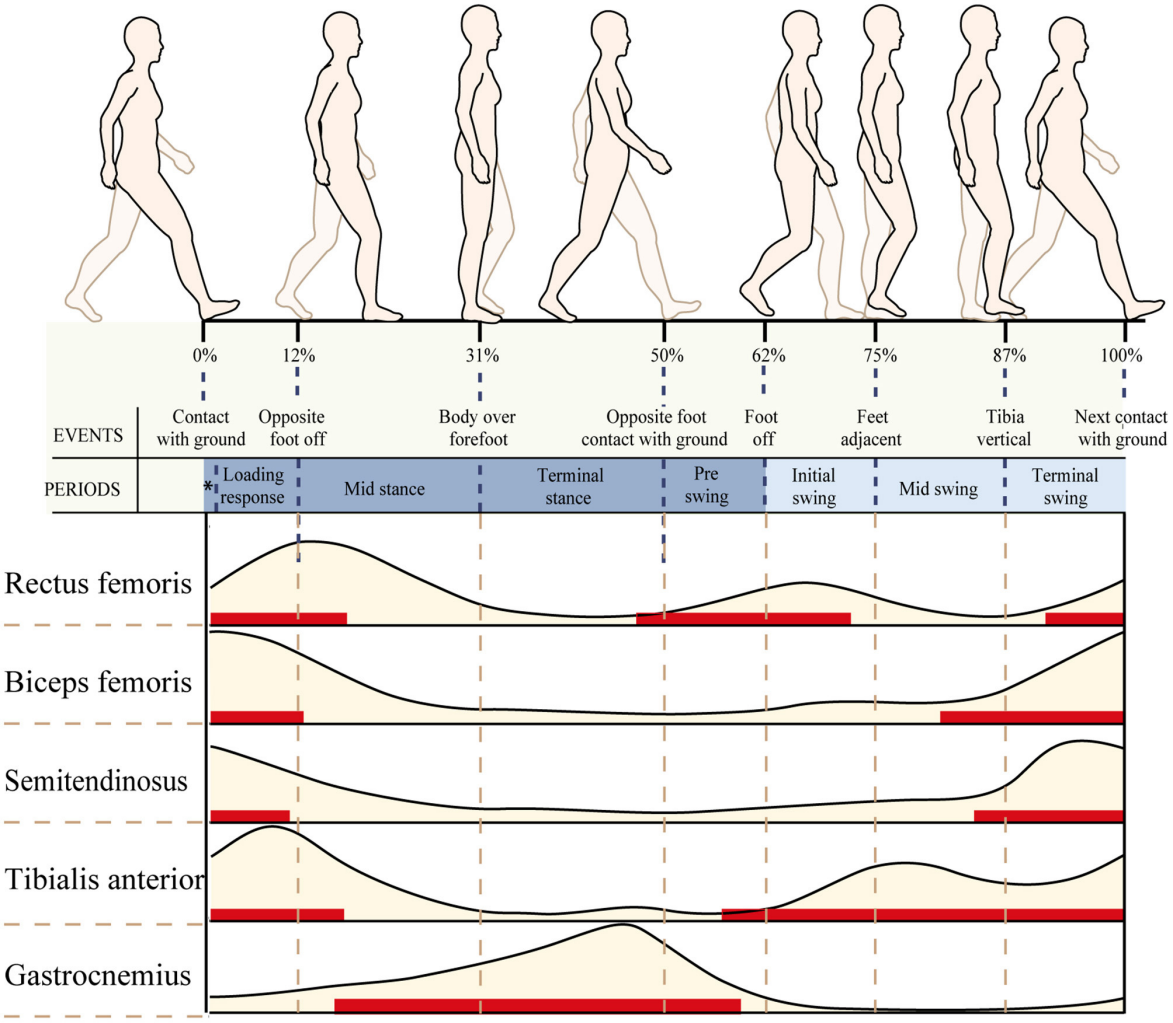
Normal electromyography (EMG) patterns for six of the major muscles in the lower extremities plotted as a function of the gait cycle. An EMG illustration showing the timing (red horizontal bars) and relative intensity (light brown shading) of muscle activation during walking.

Normal sEMG signal patterns for the major muscles in the lower extremities were plotted as a function of the gait cycle (Perc, 2005), which are shown in Figure 3. In this study, the patient's gait cycle was divided into seven subphases, and the state of the muscle was defined according to Figure 3. As shown by the red horizontal bars, the muscles contract and produce muscle force to maintain a normal gait. Muscles are thought to contract because they are activated by nervous system stimulation. So the red horizontal bars in Figure 3 indicate that the selected muscles are active during the gait cycle. In other parts during a gait cycle, the stretched muscles are passive and produce muscle tension. The RMS is used to describe the average change characteristics of sEMG signals over a period of time and refers to the RMS value of all amplitudes in this period of time. The RMS of the sEMG signals can respond to the neuromuscular function, which displays a positive correlation with muscle force and muscle tension under positive and passive conditions, respectively (Wang et al., 2011). As shown by the red horizontal bars, the RMS of the sEMG signal is proportional to muscle force. In other parts, the RMS of the sEMG signal is proportional to muscle tension.

### Statistical Analysis

The joint angle and the RMS of the sEMG signals were analyzed in this study, and paired *t*-test was used to analyze the data before and after the surgeries. All data are expressed as mean value and standard deviation of the mean (SD). The level of significance was set at *p* < 0.05, and the 95% confidence intervals (95% CIs) were calculated. The statistical analysis was performed using the SPSS Version 24 software (The Apache Software Foundation, IL, USA).

## Results

### Kinematics

The ranges of motion (ROMs) on the sagittal plane before and after the multilevel surgeries were displayed and compared in Table 2. The results indicated that the ankle ROM increased significantly after the multilevel surgeries. The ankle joint angle was more plantar flexed during the whole gait cycle.

**Table 2:** The range of motion (ROM) on the sagittal plane before and after the multilevel surgeries.

**ROM**	**Pre-operation (Deg.)**	**Post-operation (Deg.)**
Hip	45.33 ± 4.5	50.79 ± 7.0
Knee	48.32 ± 8.8	47.66 ± 12.3
Ankle^*^	23.32 ± 4.2	29.21 ± 6.8

* *Significant difference between the pre-surgery and post-surgery*.

**Figure 4:**
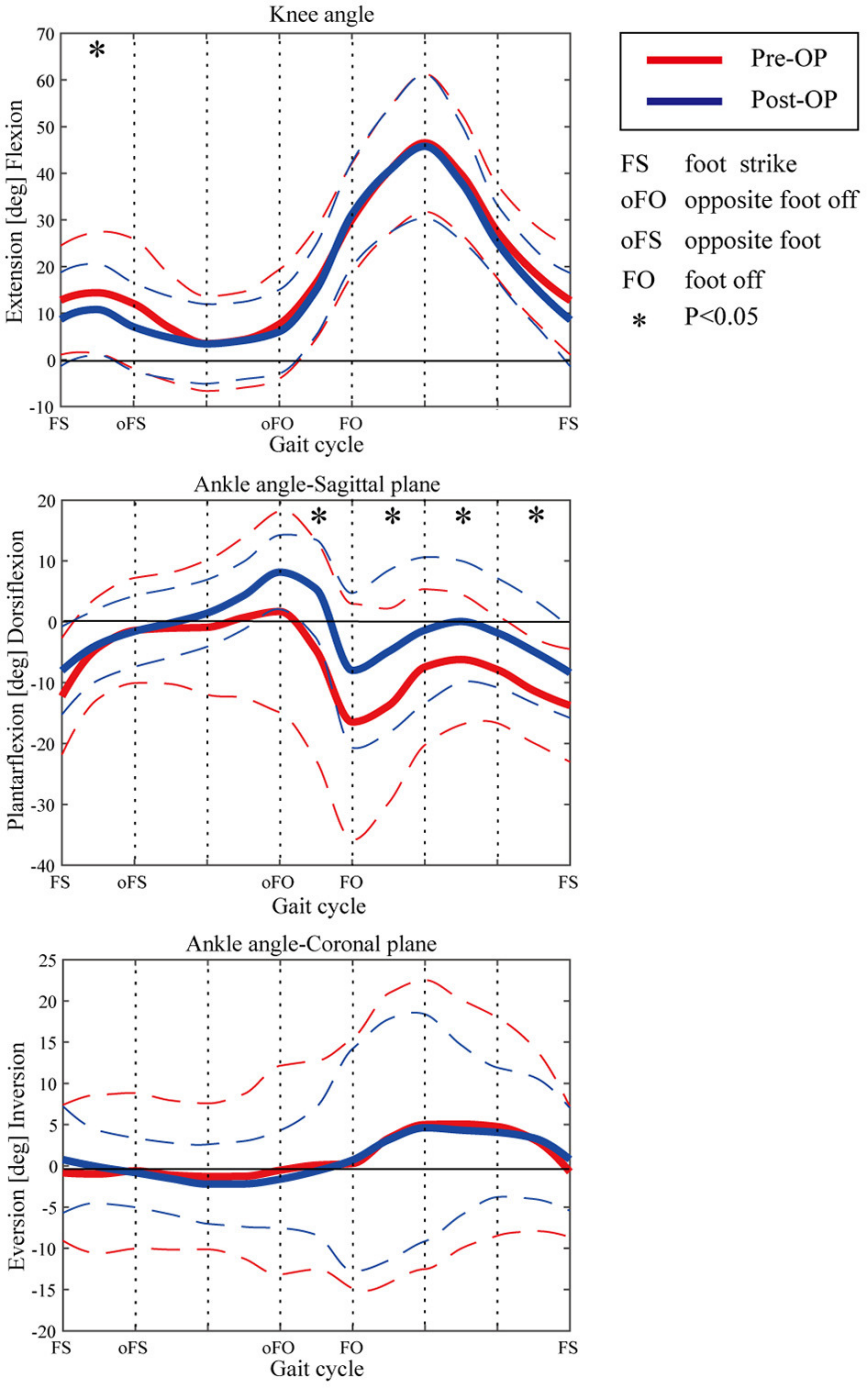
Kinematic curves of ankle and knee pre- and post-operation. Dashed lines show the standard deviation. Asterisks indicate the significant difference between the pre-surgery and post-surgery.

The kinematic parameters showed an overall improvement after surgery for the joint angles of the knee and ankle, as shown in Figure 4. Compared with those of pre-surgery, the knee flexion angle decreased significantly during the loading response (*p* < 0.05). The average knee flexion angle decreased from 20.1° to 18.3° in the whole gait cycle, and the overall knee flexion angle decreased. In the sagittal plane, the angle of ankle dorsiflexion increased significantly during the swing phase (*p* < 0.05). In terms of the ankle, the maximum dorsiflexion angle changed from 4.9° to 8.2°, and the maximum plantar flexion angle decreased from 16.5° to 8.3° in the whole gait cycle. On the coronal plane, the joint changed from the original valgus to varus during the loading response. The valgus angle became larger at the terminal stance, and the varus angle decreased significantly during the mid-swing phase.

### Electromyogram

**Figure 5:**
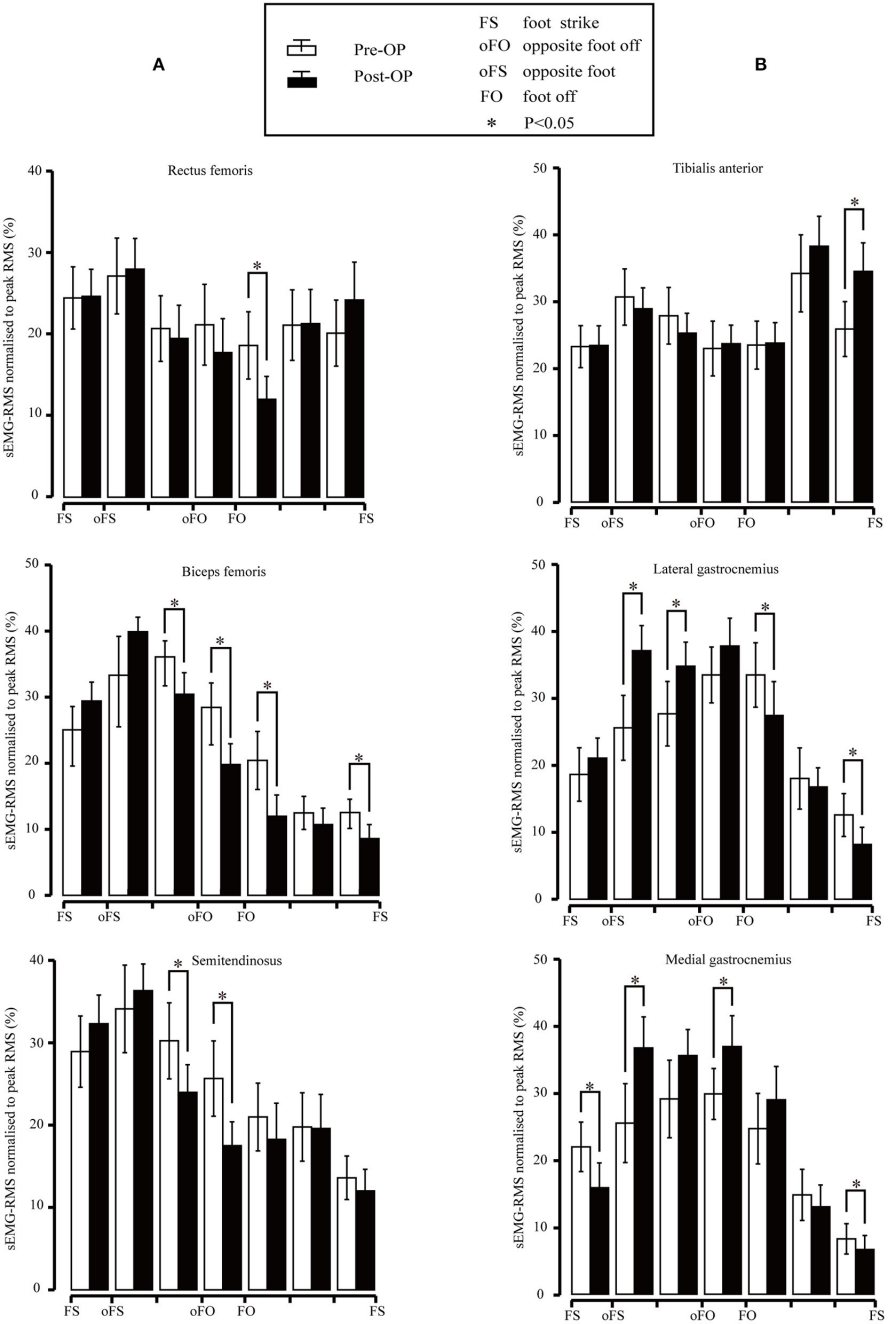
**(A)** Root mean square (RMS) values for the rectus femoris, biceps femoris, and semitendinosus surface electromyography (sEMG) signal pre- and post-surgery. Vertical lines represent 1 SD of the mean, and asterisks indicate the significant difference between the pre-surgery and post-surgery. **(B)** RMS values for the subjects of the tibialis anterior, lateral gastrocnemius, and medial gastrocnemius sEMG signal pre- and post-surgery. Vertical lines represent 1 SD of the mean, and asterisks indicate the significant difference between pre-surgery and post-surgery.

The sEMG-RMS values of the main thigh muscles before and after the surgery are presented in Figure 5A. The RMS of the rectus femoris muscle decreased significantly at the initial swing phase (*p* = 0.005). The RMS of the biceps femoris muscle decreased significantly during the terminal stance (*p* = 0.011), pre-swing (*p* = 0.01), and the initial swing phase (*p* < 0.001). We also observed a statistically significant decrease during the terminal swing (*p* = 0.02). The difference between several subphases was that the biceps femoris should be active during the terminal swing. The RMS of the semitendinosus muscle decreased significantly during the terminal stance (*p* = 0.016) and pre-swing (*p* = 0.002).

The sEMG-RMS values of the tibialis anterior, lateral gastrocnemius, and medial gastrocnemius muscles in the gait cycle before and after the surgeries are compared and illustrated in Figure 5B. Results revealed that the RMS of the tibial anterior muscle was significantly higher after the surgery than before during terminal swing (*p* = 0.003). In addition, the RMS of the lateral gastrocnemius muscle was significantly higher pre-surgery during mid-stance (*p* = 0.001) and terminal stance (*p* = 0.034); in contrast, it became significantly lower compared to before surgery during the initial swing (*p* = 0.028) and terminal swing (*p* < 0.001). The medial gastrocnemius muscle showed significant improvement compared to that of pre-surgery during mid-stance (*p* = 0.005) and pre-swing (*p* = 0.019); nevertheless, the RMS significantly decreased during the loading response (*p* = 0.009) and terminal swing (*p* = 0.021).

Based on Figure 3, the sEMG-RMS changes of muscle activities were transferred to the adjustments of muscle tension and force before and after the surgery during the gait cycle, displayed on Table 3. For the thigh muscles, the changes in the flexors were greater than the extensors. For the calf muscles, the changes in the plantar flexors were greater than those in the dorsiflexors. These showed that the changes in the muscles of the posterior side of the lower limbs are greater than those of the anterior side. It can be seen that muscle tension decreased, and some muscle forces increased after the surgery for most muscles. The muscle tension of the biceps femoris and semitendinosus muscles was reduced at the terminal stance, pre–swing, and initial swing. The medial gastrocnemius muscle tension during the loading response and terminal swing was significantly reduced. The lateral gastrocnemius muscle tension during the initial and terminal swing was significantly reduced. But the eccentric contraction muscle force of the gastrocnemius muscles increased in the stance phase. At the terminal swing, the force of the tibial anterior muscle improved. On the contrary, the muscle force of the rectus femoris and biceps femoris was reduced during the initial swing and terminal swing, respectively.

**Table 3:** The change of muscle function before and after the multilevel surgeries.

**Muscles**	**Loading response**	**Mid-stance**	**Terminal stance**	**Pre-swing**	**Initial swing**	**Mid-swing**	**Terminal swing**
Rectus femoris	/	/	/	/	MFD	/	/
Biceps femoris	/	/	MTD	MTD	MTD	/	MFD
Semitendinosus	/	/	MTD	MTD	/	/	/
Tibialis anterior	/	/	/	/	/	/	MFI
Lateral gastrocnemius	/	MFI	MFI	/	MTD	/	MTD
Medial gastrocnemius	MTD	MFI	/	MFI	/	/	MTD

*MFI, muscle force increase; MFD, muscle force decrease; MTD, muscle tension decrease*.

## Discussion

The realization of every movement depends on the nervous system to regulate the coordinated activities of related muscle groups so as to complete a normal gait when walking (Lieber, 2002). Patients with neurological diseases also have obstacles in their muscle co-contraction function (Banks et al., 2017; Zhixian et al., 2018). Patikas et al. (2007) proved that the sEMG signal changed after multilevel surgeries during walking, but they did not explain the changes in neuromuscular function. The RMS of the sEMG signal in the passive state proved that the muscle tension decreased after selective femoral neurotomy, but we could not know the change of neuromuscular function during walking (Wang et al., 2011). Taking into consideration normal sEMG signal patterns for the major muscle, the muscles can be divided into passive and active movements (Perc, 2005). By studying the change of the RMS of the sEMG signal, we found that muscle force increased and muscle tension decreased. This study proves that sEMG signals can be used to evaluate neuromuscular function during walking.

The changes of muscle force and tension after multilevel surgeries had a good effect in children with CP. During the loading response, the gastrocnemius muscle tension at initial contact may result in a stretch reflex response. The tensed gastrocnemius muscle does not allow the knee to fully extend during the initial contact (Hullin et al., 1996). The gastrocnemius muscle tension during the loading response was significantly reduced from Table 3, which helped the knee joint to get the maximum extension when the heel is on the ground. In the stance phase, the eccentric contraction muscle force of the gastrocnemius muscles increased, which could control the calf leaning forward, and generated the driving force for the human body to move forward. Coupled with the increase in ankle dorsiflexion, it helped to increase the power of the plantar flexors, which was very important for the body to move forward (Winter, 1983). The muscle tension of the biceps femoris and semitendinosus muscles is reduced. At this time, the knee joint was passively flexed and the lowered knee flexor muscle tension can relieve the burden of the patient's knee flexion on the knee joint (Nardo et al., 2017). In the swing phase, the muscle tension of the gastrocnemius muscles was reduced, which was conducive to the ankle joint from plantar flexion to dorsiflexion, so the patient's ankle dorsiflexion was improved. The reduced gastrocnemius muscle tension could also make the leg swing from fast to slow to make the heel land smoothly. At the terminal swing, the increase of tibial anterior muscle force could improve the ankle dorsiflexion ability, prevent foot drop, and push forward (Agarwal et al., 2020a). Unfortunately, the muscle force of the rectus femoris and biceps femoris was reduced during the initial swing and terminal swing, respectively. Biceps femoris muscle force can help to coordinate with the coordinated contraction of the rectus femoris to slow down the forward swinging calf and prepare for the heel landing, but the eccentric contraction muscle force decreased, which was not conducive to the recovery of normal gait posture for children with CP.

Children with CP mainly manifested as crouching gait and clubfoot. The crouching gait is specifically manifested that the knee flexion angle is too large, and the patient cannot walk upright. Long-term crouching posture could lead to knee cartilage degradation and joint pain (O'Sullivan et al., 2020). The outcomes of this study demonstrated that the knee flexion deformity of the patient had improved after surgery. Clubfoot is clinically manifested as restricted ankle dorsiflexion ability and foot varus. Flaccidity of foot and foot varus could cause the sole of the foot not to effectively touch the ground, and the body center is unable to move forward effectively, which resulted in walking dysfunction (Agarwal et al., 2020b). Through surgery, the patient's ankle dorsiflexion had been greatly improved. On the coronal plane, the condition of the foot varus was also relieved. It was worth mentioning that during the loading response, the foot changed from varus to valgus, which was exactly in line with the law of normal gait. The normal subtalar joint movement of the human body during the stance phase is: a slight supination in the early and middle stages, then pronation rapidly, and supination again in the middle and late stages (Neumann, 2010).

## Conclusion

This article presented the assessments of multilevel surgical treatment effects in children with SCP by investigating the changes in sEMG signal patterns pre- and post-surgeries. By extracting the RMS of the sEMG signal and muscle activation state, the change of the RMS is transformed into the change of muscle force and muscle tension. After multilevel surgeries, for calf muscles, the muscle force increased and muscle tension decreased. Both the muscle force and muscle tension of thigh muscle decreased. This reduced muscle force could be compensated for by rehabilitation training. In conclusion, this is consistent with the operation principle that FSPR can reduce the muscle tension of the lower limb muscles, and the tibial anterior muscle transfer surgery is thought to balance the muscle force of the calf muscles. Therefore, the findings of the present study support that the RMS of the sEMG signal can describe neuromuscular function of the patients during walking, and the multilevel surgeries are feasible and effective to treat SCP.

In this study, we employed the muscle activation state during the walking cycle to establish the relationship between the RMS of the sEMG signal and muscle force and tension. Because the muscle activation states of CP are different from those of normal people, our subsequent work will focus on the muscle activation state of children with CP during walking. By dividing the muscle activation state of children with CP, the expected findings of future studies would be more meaningful and quantitative to the surgical treatments for CP.

## Data Availability Statement

The raw data supporting the conclusions of this article will be made available by the authors, without undue reservation.

## Ethics Statement

The studies involving human participants were reviewed and approved by Ethical code from Shanghai University of Medicine and Health Sciences: 2019-ZYXM1-04−420300197109053525. Written informed consent to participate in this study was provided by the participants' legal guardian/next of kin.

## Author Contributions

SL, XL, and SZ conceived and designed the experiments, analyzed and interpreted the data, and wrote the manuscript. SZ, YT, JS, and QM performed the experiments and wrote the manuscript. HY and CS designed, interpreted the data, and revised the manuscript. All authors contributed to the article and approved the submitted version.

## Conflict of Interest

The authors declare that the research was conducted in the absence of any commercial or financial relationships that could be construed as a potential conflict of interest.

## References

[B1] Agarwal A. Gourav J. Neeraj G. (2020a). Comparison of three different methods of anterior tibial tendon transfer for relapsed clubfoot: a pilot study. J. Clin. Orthopaed. Trauma. 11, 240–244. 10.1016/j.jcot.2018.09.001 32099287 PMC7026582

[B2] Agarwal A. Gupta S. Sud A. Agarwal S. (2020b). Results of modified ponseti technique in difficult clubfoot and a review of literature. J. Clin. Orthopaed. Trauma 11, 222–231. 10.1016/j.jcot.2019.05.003 32099284 PMC7026550

[B3] Banks C. L. Huang H. J. Little V. L. Patten C. (2017). Electromyography exposes heterogeneity in muscle co-contraction following stroke. Front. Neurol. 8:699. 10.3389/fneur.2017.00699 29312124 PMC5743661

[B4] Bell K. J. Õunpuu S. DeLuca P. A. Romness M. J. (2002). Natural progression of gait in children with cerebral palsy. J. Pediatr. Orthop. 22, 677–682. 10.1097/01241398-200209000-00020 12198474

[B5] Buddhdev P. Fry N. R. Lepage R. Wiley M. Gough M. Shortland A. P. (2017). Abnormality of standing posture improves in patients with bilateral spastic cerebral palsy following lower limb surgery. Gait Post. 54:255. 10.1016/j.gaitpost.2017.03.014 28371738

[B6] Buurke J. H. Hermens H. J. Roetenberg D. Harlaar J. Rosenbaum D. Kleissen R. F. M. (2004). Influence of hamstring lengthening on muscle activation timing. Gait Post. 20, 48–53. 10.1016/S0966-6362(03)00092-4 15196520

[B7] Campanini I. Disselhorst-Klug C. Rymer W. Z. Merletti R. (2020). Surface EMG in clinical assessment and neurorehabilitation: barriers limiting its use. Front. Neurol. 11:934. 10.3389/fneur.2020.00934 32982942 PMC7492208

[B8] Cappellini G. Sylos-Labini F. Assenza C. Libernini L. Ivanenko Y. (2020). Clinical relevance of state-of-the-art analysis of surface electromyography in cerebral palsy. Front. Neurol. 11:583296. 10.3389/fneur.2020.583296 33362693 PMC7759523

[B9] Chin E. M. Gwynn H. E. Robinson S. Hoon A. H. (2020). Principles of medical and surgical treatment of cerebral palsy. Neurol. Clin. 38, 397–416. 10.1016/j.ncl.2020.01.009 32279717 PMC7158771

[B10] Crenna P. (1998). Spasticity and spastic gait in children with cerebral palsy. Neurosci. Biobehav. Rev. 22:571. 10.1016/S0149-7634(97)00046-8 9595571

[B11] Daly C. Lafferty E. Joyce M. Malone A. (2019). Determining the most effective exercise for gluteal muscle activation in children with cerebral palsy using surface electromyography. Gait Post. 70:270. 10.1016/j.gaitpost.2019.03.013 30913506

[B12] Domagalska–Szopa M. Szopa A. (2019). Gait pattern differences among children with bilateral cerebral palsy. Front. Neurol. 10:183. 10.3389/fneur.2019.00183 30930827 PMC6423305

[B13] El Batti S. Solla F. Clément J. L. Rosello O. Oborocianu I. Chau E. Rampal V. (2016). Initial treatment of congenital idiopathic clubfoot: prognostic factors. Orthopaed. Traumatol. Surg. Res. 102, 1081–1085. 10.1016/j.otsr.2016.07.012 27765520

[B14] El-Fadl A. Mahmoud S. (2013). An unusual aberrant muscle in congenital clubfoot: an intraoperative finding. J. Foot Ankle Surg. 52, 380–382. 10.1053/j.jfas.2012.12.012 23415495

[B15] Farina D. Merletti R. Enoka R. M. (2004). The extraction of neural strategies from the surface EMG. J. Appl. Physiol. 96, 1486–1495. 10.1152/japplphysiol.01070.2003 15016793

[B16] Fox M. D. Reinbolt J. A. ÕUnpuu S. Delp S. L. (2009). Mechanisms of improved knee flexion after rectus femoris transfer surgery. J. Biomech. 42, 614–619. 10.1016/j.jbiomech.2008.12.007 19217109 PMC2929172

[B17] Gage J. R. Novacheck T. F. (2001). An update on the treatment of gait problems in cerebral palsy. J. Pediatr. Orthop. 10, 265–274. 10.1097/00009957-200110000-00001 11727367

[B18] Gagnat Y. Brndvik S. M. Roeleveld K. (2020). Surface electromyography normalization affects the interpretation of muscle activity and coactivation in children with cerebral palsy during walking. Front. Neurol. 11:202. 10.3389/fneur.2020.00202 32362862 PMC7180206

[B19] Graham D. Aquilina K. Cawker S. Paget S. Wimalasundera N. (2016). Single-level selective dorsal rhizotomy for spastic cerebral palsy. J. Spine Surg. 2:195. 10.21037/jss.2016.08.08 27757432 PMC5067263

[B20] Granata K. P. Darin A. P. Mark F. A. (2005). Repeatability of surface EMG during gait in children. Gait Post. 22, 346–350. 10.1016/j.gaitpost.2004.11.014 16274917 PMC1628350

[B21] Hullin M. G. Robb J. E. Loudon I. R. (1996). Gait patterns in children with hemiplegic spastic cerebral palsy. J. Pediatr. Orthopaed. 5, 247–251. 10.1097/01202412-199605040-00006 8897257

[B22] Kapti A. O. (2014). Dynamic simulation of tibialis posterior tendon transfer in the treatment of drop-foot. Biocybernet. Biomed. Eng. 34, 132–138. 10.1016/j.bbe.2014.01.001

[B23] Lauer R. T. Smith B. T. Shewokis P. A. Mccarthy J. J. Tucker C. A. (2007). Time–frequency changes in electromyographic signals after hamstring lengthening surgery in children with cerebral palsy. J. Biomech. 40, 2738–2743. 10.1016/j.jbiomech.2007.01.001 17328900

[B24] Lieber R. L. (2002). Skeletal Muscle Structure, Function, and Plasticity. Baltimore: Lippincott Williams & Wilkins.

[B25] Nardo F. D. Strazza A. Mengarelli A. Cardarelli S. Tigrini A. Verdini F.. (2019). Emg-based characterization of walking asymmetry in children with mild hemiplegic cerebral palsy. Biosensors 9:82. 10.3390/bios9030082 31252517 PMC6784376

[B26] Nardo F. D. Strazza A. Mengarelli A. Ercolani S. Fioretti S. (2017). Surface EMG patterns for quantification of thigh muscle co-contraction in school-age children: normative data during walking. Gait Post. 61, 25–33. 10.1016/j.gaitpost.2017.12.025 29294416

[B27] Neumann D. A. (2010). Kinesiology of the Musculoskeletal System; Foundation for Rehabilitation. St. Louis: Mosby & Elsevier.

[B28] Onishi H. Yagi R. Akasaka K. Momose K. Ihashi K. Handa Y. (2000). Relationship between EMG signals and force in human vastus lateralis muscle using multiple bipolar wire electrodes. J. Electromyogr. Kinesiol. 10, 59–67. 10.1016/S1050-6411(99)00020-6 10659450

[B29] O'Sullivan R. A. Marron A. Brady K. (2020). Crouch gait or Flexed-knee gait in cerebral palsy; is there a difference? A systematic review. Gait Post. 81:233. 10.1016/j.gaitpost.2020.09.001 32927222

[B30] Parent A. Pouliot-Laforte A. Maso F. D. Cherni Y. Ballaz L. (2019). Muscle fatigue during a short walking exercise in children with cerebral palsy who walk in a crouch gait. Gait Post. 72:22–7. 10.1016/j.gaitpost.2019.05.021 31132593

[B31] Patikas D. Wolf S. Döderlein L. (2005). Electromyographic evaluation of the sound and involved side during gait of spastic hemiplegic children with cerebral palsy. Eur. J Neurol. 12, 691–699. 10.1111/j.1468-1331.2005.01047.x 16128870

[B32] Patikas D. Wolf S. I. Schuster W. Armbrust P. Dreher T. Döderlein L. (2007). Electromyographic patterns in children with cerebral palsy: do they change after surgery? Gait Post. 26, 362–371. 10.1016/j.gaitpost.2006.10.012 17140796

[B33] Perc M. (2005). The dynamics of human gait. Eur. J. Phys. 26:525. 10.1088/0143-0807/26/3/017

[B34] Perry J. Davids J. R. (1992). Gait analysis: normal and pathological function. J. Pediatr. Orthop. 12:815. 10.1097/01241398-199211000-00023

[B35] Qijia Z. Liang T. Yanyan W. Bo X. Min S. (2019). Feasibility and effectiveness of a newly modified protocol-guided selective dorsal rhizotomy via single-level approach to treat spastic hemiplegia in pediatric cases with cerebral palsy. Child's Nerv. Syst. 35, 2171–2178. 10.1007/s00381-019-04194-0 31144022

[B36] Turner R. P. (2009). Neurophysiologic intraoperative monitoring during selective dorsal rhizotomy. J. Clin. Neurophysiol. 26:82. 10.1097/WNP.0b013e31819f9077 19279497

[B37] Wang S. Miao S. Zhuang P. Chen Y. Liu H. Zuo H. (2011). Assessment of surface electromyographic clinical analysis of selective femoral neurotomy on cerebral palsy with stiff knee. J. Neurosci. Methods 199, 98–102. 10.1016/j.jneumeth.2011.04.031 21554900

[B38] Winter D. A. (1983). Energy generation and absorption at the ankle and knee during fast, natural, and slow cadences. Clin. Orthopaed. Relat. Res. 175, 147–154. 10.1097/00003086-198305000-00021 6839580

[B39] Winters T. F. Gage J. R. Hicks R. R. (1987). Gait patterns in spastic hemiplegia in children and adults. J. Bone Joint Surg. 69, 437–441. 10.2106/00004623-198769030-00016 3818706

[B40] Zhixian G. Lin C. Qiliang X. Nong X. Wei J. (2018). Degraded synergistic recruitment of semg oscillations for cerebral palsy infants crawling. Front. Neurol. 9:760. 10.3389/fneur.2018.00760 30279674 PMC6153367

